# Policy Makes Politics: Comment on "Modelling the Health Policy Process: One Size Fits All or Horses for Courses?"

**DOI:** 10.34172/ijhpm.2023.8073

**Published:** 2023-09-17

**Authors:** Scott L. Greer

**Affiliations:** ^1^Health Management and Policy, Global Public Health, and Political Science, University of Michigan-Ann Arbor, Ann Arbor, MI, USA; ^2^European Observatory on Health Systems and Policies, Brussels, Belgium

**Keywords:** Politics, Health Policy, Public Policy, Health Policy Process

## Abstract

Powell and Mannion’s review of reviews maps the landscape of health policy research, showing a number of problematic and longstanding features. This commentary focuses on the extent to which health parochialism is good for the scientific development of the literature, the extent to which a "tournament of theories" actually develops our understanding of health policy process, and, finally, whether circumscribed theories of the policy process might be missing some of the most important and useful findings of broader comparative politics, which focus on the ways policies create politics over time. It concludes that health parochialism and focus on a circumscribed policy process is not likely to be helpful because it distracts attention from the ways in which coalitions and institutions over time shape politics and policy, a finding explored by scholars of many sectors whose findings should influence health policy research.

 One regular theme in scholarly writing on health politics is complaint about the handling of politics and policy in broader health literature. There is complaint about the limited extent and quality many writers see in both scholarship on health policy-making. And there is complaint about the ways in which the putatively apolitical and technical discourses of medicine and public health disguise political interests, foster naivete, and obscure causal mechanisms that might be affected by political action. When policy-making is taught, it is often either oversimplified, or uses approaches that outsiders to the health world might regard as reinventions of the wheel. Even researchers and policy-makers who are privately very sensitive to politics and astute in political analysis will often be blandly technocratic and seemingly naïve in public.

 Powell and Mannion^1^ address this issue by looking at reviews of the theories of the policy process that are used in health. These articles are reviews of the choice and deployment of theory in health policy articles and their number means that we can be more confident that individual review authors’ choices are not biasing results.

 The results are probably unsurprising to people versed in the literature, for all that it is nice to see confirmation that they aren’t just impressions born of attending particular conferences or reading particular journals. Powell and Mannion find a large number of articles with what amounts to repeated uses of off-the-shelf theories. They also find a dismayingly large number coded by team after team as not really having a theory of the policy process at all. It is one thing, from the perspective of science, to have repeated tests of a well-known theory, which is what Mannion and Powell appear to find in the literature. That can be valuable and contribute to incremental progress. It is another thing to have articles with no theory at all. What are we supposed to learn from them? That is probably, then, the first significant problem — lack of progress on the problem that Walt and Gilson identified, of too much attention to the content of policy rather than the actors, processes, and context.^2^

 There are, however, three other issues raised in the article that merit attention. The first issue, foregrounded in Powell and Mannion’s analysis, is health policy parochialism, notably as seen in theories only found in health policy scholarship. We could speculate on reasons why this parochialism exists. Perhaps health policy researchers have found other debates and topics, such as the social determinants of health, more interesting and productive (could the authors of the articles coded as atheoretical be engaged in those debates?). Perhaps the world of health policy research is so big and well-funded that it can afford to have theories, such as the “health policy analysis triangle” that are unknown outside health policy. It might also be that health policy research is so heavily influenced by the money and power in academic medicine as to introduce distortions. A subtle theory of public policy is likely to far exceed medical or public health journal word counts and citation mechanisms, let alone the patience of referees and editors; funders tend to support work on the content of policies, lots of people want to see “impact,” defined as adoption of a policy or intervention rather than understanding of the process, and it is noteworthy to see an educational program in most areas of biomedicine which has more than one class on the whole of “policy.” This is an ecology of knowledge that might reward the overapplication of very simple theories, and not support the development and testing of more subtle theories as we see in social sciences. The tendency of medical and public health journals to publish what looks like political science and policy analysis in commentary or opinion sections hardly helps by mixing what might be sophisticated policy analysis with senior doctors’ opinions or organizational manifestos. These are just speculative guesses but might add up to what Powell and Mannion report.

 It does seem that health policy research publication is more susceptible than other areas of policy analysis to producing very simple renderings of social science concepts and then mistaking them for complete theories. As Powell and Mannion show, John Kingdon’s theory, known as the multiple streams perspective, is perhaps a good example. An unarguably powerful and parsimonious theory, it can still be be distorted in applications that ignore its difficulties and over-emphasize the policy stream where most public health researchers and students instinctively locate themselves.^3^

 The second issue is how much scholarly progress is possible through the simple application of well-known theories. Mere use of an established theoretical framework is not necessarily an indicator of theoretical progress since repeatedly testing the applicability or explanatory power of a good theory can leave us in an endless, inconclusive, tournament of theories. The difference between, for example, the multiple streams approach and the advocacy coalition approach lies not in anomalies or limited coverage; they can both explain a vast swathe of health policy. The difference lies in what they foreground, with implications for action and further analysis. Simply showing that they can describe and explain with reliability does not show us which ones merit further development and use. If every public policy theory is as good as every other one, then we should worry about the scope for future theoretical development and its attractiveness to new researchers.

 The third issue is the question of whether theories of the health policy process are studying the right thing at all. Theories of “The policy process” tend to focus on specific pieces of legislation or on characterizing the process which shapes their likelihood of introduction and adoption. As the article notes, there is less attention in the literature that they review to the whole complex of post-adoption politics or to feedback loops in which one policy decision reshapes policy options and political coalitions for the future. These policy legacies and feedbacks go far beyond implementation (in itself a difficult issue) because they change what is possible. That means that even when they appear, circumscribing them to theories of the policy process removes much of their power and generative potential.

 The problem that is obscured by a focus on the policy process is that policy creates politics.^4^ The ways in which policy creates politics have been a major issue in the analysis of how health systems develop, with decisions taken in the early twentieth century on issues such as the role of employers and the organized medical profession shaping what was politically and practically possible later (this literature is voluminous, but usefully discussed in the course of Tuohy’s important contribution).^5^ More contemporaneous developments, for example the politics of universal health coverage in middle-income countries such as Brazil and Thailand, likewise tend to be explained by politics over time and path dependency.^6^ These are dynamics that are difficult to capture in any “theory of the policy process.”

 Policies create politics by creating institutions which can be sticky, by creating coalitions of beneficiaries and state capacity, and by raising the costs of alternative options.^7^ Policies and legacies can be more important than formal political institutions. In federations, for example, the structure of healthcare finance and delivery can be stickier and do more to shape money and power among governments than formal constitutional law.^8^

 The question this literature raises is of how policies become entrenched. Broadly, answers fall into two categories. One is older and grew out of the “historical institutionalist” tradition in comparative politics. It focuses on the ways in which institutions, including policies, shape the rules of the game and the costs of change. Its key concepts are punctuated equilibrium (seen in the scholarship Powell and Mannion review) and path dependency but it has developed a much more elaborate and contested theoretical language for understanding the change.^9^

 The other broad strand focuses more on coalitions, putting more focus on alliances of interests and less on the tendency of institutions and policies to persist.^7,10^ Who benefits from a policy and what degrees of freedom exist to create new, and different, coalitions? The creation of coalitions can include the creation of identities by politics, eg, by making people aware of a benefit that they are receiving and organizing them around it. This literature has produced some rules of thumb for policy. For example, it suggests emphasizing visibility and simplicity so that beneficiaries understand that they are getting something from the policy, can reward the politicians who created it, and can be alerted to attacks on it.^11,12^ Perhaps the best cases of this logic are National Health Service systems in the United Kingdom, which despite their complexity have clear public profiles and a public that is generally unwilling to support attacks on them.^13^ It is worth noting that making benefits obvious and simple so that beneficiaries appreciate them might seem like basic politics but intelligent and skilled politicians have often preferred complicated or deliberately obscure schemes, eg, “nudge” policies or tax credits.

 A narrow focus on “the policy process” is not unique to researchers, of course. Policy-makers themselves can have understandably short time horizons or be so focused on optimal policy design and legislative politics that they fail to consider policy feedbacks and the ways that their own policies will build or undermine sustainable supporting coalitions. One article on the United States’ 2010 Affordable Care Act carried the memorable, and accurate, subtitle “Democratic Policy Makers Overlooked Implementation, Post-Enactment Politics, and Policy Feedback Effects.”^14^ Even when there was sophisticated thinking about how implementation would work, it seemed to not extend past the next elections.^15^ This suggests that there is some scope for very practical crossover between political science and even sophisticated political actors who can discuss the ways to entrench desired policies by building sticky institutions and stable coalitions of supporters with resources that will enable the policy to survive changes of government and conditions.

 Powell and Mannion conclude by suggesting that there are two reasonable ways to go in the future: either building a specific analytical language for health politics and policy, or by engaging with the broader debates about policy-making and change. It stands to reason that drawing on multiple areas of public policy and political research would increase the odds of strong and parsimonious theoretical claims emerging, while the risk of having health-specific theories would be the creation of an echo chamber that would not improve explanations over time. The answer to their question should primarily depend, though, on the value of the approaches in producing better explanations of events in health policy. If comparative politics scholarship takes us beyond “theories of the policy process” into a broader conversation about policy design, entrenchment, and political sustainability, then we should support more dialogue with comparative politics and other fields. Health policy research might not, as they argue, have assimilated Walt and Gilson’s injunction to focus more on actors, processes, and context, but it might nevertheless be time to add an injunction to think about how policy makes politics.

## Ethical issues

 Not applicable.

## Competing interests

 Author declares that he has no competing interests.
